# Supplementary material for: Mechanisms governing the pioneering and redistribution capabilities of the non-classical pioneer PU.1
Source: Nat Commun. 2020 Jan 21;11:402. doi: 10.1038/s41467-019-13960-2 (PMC6972792; doi:10.1038/s41467-019-13960-2)
Supplement: Supplementary file 7 — Source data [file 41467_2019_13960_MOESM7_ESM.zip › Source_Data/FigureS2/FigureS2B_MotifScanOutput/KmeansCluster6/homerResults/motif8.similar.html]

motif8

## Information for motif8

T
C
A
G
C
T
G
A
G
A
C
T
C
A
G
T
T
C
A
G
G
A
C
T
T
C
G
A
C
G
T
A
T
C
A
G
G
C
A
T
A
G
C
T
G
A
C
T
A
G
T
C
G
A
T
C
  
Reverse Opposite:  

C
T
A
G
C
T
A
G
C
T
G
A
C
T
G
A
C
G
T
A
A
G
T
C
C
G
A
T
A
C
G
T
C
T
G
A
A
G
T
C
G
C
T
A
C
T
G
A
G
A
C
T
A
G
T
C
  

|  |  |
| --- | --- |
| p-value: | 1e-78 |
| log p-value: | -1.818e+02 |
| Information Content per bp: | 1.756 |
| Number of Target Sequences with motif | 143.0 |
| Percentage of Target Sequences with motif | 4.34% |
| Number of Background Sequences with motif | 167.0 |
| Percentage of Background Sequences with motif | 0.39% |
| Average Position of motif in Targets | 86.6 +/- 72.3bp |
| Average Position of motif in Background | 96.2 +/- 71.8bp |
| Strand Bias (log2 ratio + to - strand density) | -0.6 |
| Multiplicity (# of sites on avg that occur together) | 1.00 |
| Motif File: | file (matrix) reverse opposite |

### Similar de novo motifs found

|  |  |  |  |  |  |  |  |
| --- | --- | --- | --- | --- | --- | --- | --- |
| Rank | Match Score | Redundant Motif | P-value | log P-value | % of Targets | % of Background | Motif file |
| 1 | 0.968 | C T A G C T G A G A C T A G C T T A C G G C A T T C G A C T G A T C A G A C G T C G A T A G C T G A T C | 1e-74 | -172.609072 | 4.40% | 0.45% | motif file (matrix) |
| 2 | 0.944 | C T G A G A C T A G C T T A C G G C A T T C G A C G T A T C A G A C G T G A C T A G C T G A T C | 1e-70 | -161.978855 | 3.95% | 0.37% | motif file (matrix) |
| 3 | 0.874 | C T A G C T G A G C A T A C G T T A C G G C A T T C G A C G T A C T A G G C A T G A C T | 1e-63 | -145.764026 | 4.52% | 0.63% | motif file (matrix) |
| 4 | 0.785 | C G A T C G T A C G T A A C T G A C G T C G A T A G C T G T A C G A T C A G C T | 1e-46 | -108.130498 | 4.25% | 0.81% | motif file (matrix) |
| 5 | 0.794 | A C G T C G T A C G T A A C T G A C G T C G A T A C G T A G T C | 1e-29 | -68.846969 | 3.77% | 1.00% | motif file (matrix) |
